# Latent class analysis to identify subphenotypes predicting pediatric splenic pseudoaneurysm following blunt spleen injuries: A post-hoc analysis

**DOI:** 10.2478/jccm-2025-0037

**Published:** 2025-10-31

**Authors:** Yuki Kishihara, Hideto Yasuda, Morihiro Katsura, Masahiro Kashiura, Shunsuke Amagasa, Yutaro Shinzato, Yutaka Kondo, Shigeki Kushimoto, Takashi Moriya

**Affiliations:** Department of Emergency and Critical Care Medicine, Jichi Medical University Saitama Medical Center, Saitama, Japan; Department of Clinical Research Education and Training Unit, Keio University Hospital Clinical and Translational Research Center, Tokyo, Japan; School of Nursing, Midwifery and Social Work, UQ Centre for Clinical Research, The University of Queensland,Australia; School of Nursing and Midwifery; Alliance for Vascular Access Teaching and Research, Griffith University,Australia; Department of Surgery, Okinawa Chubu Hospital, Okinawa, Japan; Department of Emergency and Transport Medicine, National Center for Child Health and Development, Tokyo, Japan; Department of Emergency and Disaster Medicine, Juntendo University Graduate School of Medicine, Tokyo, Japan; Division of Emergency and Critical Care Medicine, Tohoku University Graduate School of Medicine, Miyagi, Japan

**Keywords:** false aneurysm, latent class analysis, pediatrics, spleen

## Abstract

**Aim of the study:**

The rupture of delayed formed splenic pseudoaneurysms after pediatric blunt splenic injuries undergoing nonoperative management (NOM) can be life-threatening. We aimed to identify the sub-phenotypes predicting delayed splenic pseudoaneurysm formation following pediatric blunt splenic injury using latent class analysis (LCA).

**Material and Methods:**

In this retrospective observational study conducted using a multicenter cohort of pediatric trauma patients, we included pediatric patients (aged ≤16 years) who sustained blunt splenic injuries and underwent NOM from 2008 to 2019. LCA was performed using clinically important variables, and 2–5 sub-phenotypes were identified. The optimal number of sub-phenotypes was determined on the basis of clinical importance and Bayesian information criterion. The association between sub-phenotyping and delayed splenic pseudoaneurysm formation was analyzed using univariate logistic regression analysis with odds ratios (ORs) and 95% confidence intervals (CIs).

**Results:**

The LCA included 434 patients and identified three optimal sub-phenotypes. Contrast extravasation (CE) of initial CT in the spleen was observed in 22 patients (68.8%) in Sub-phenotype 1, 49 patients (25.7%) in Sub-phenotype 2, and 22 patients (10.4%) in Sub-phenotype 3 (p = 0.007). Delayed splenic pseudoaneurysm was observed in 46 patients (10.6%), including seven patients (21.9%) in Sub-phenotype 1, 25 patients (13.1%) in Sub-phenotype 2, and 14 patients (6.6%) in Sub-phenotype 3 (p = 0.01). Logistic regression analysis for delayed splenic pseudoaneurysm formation using Sub-phenotype 3 as the reference revealed an OR (95% CI) of 3.94 (1.45–10.7) in Sub-phenotype 1 and 2.12 (1.07–4.21) in Sub-phenotype 2.

**Conclusions:**

The LCA identified three sub-phenotypes showing statistically significant differences for delayed splenic pseudoaneurysm formation. Our findings suggest that cases with CE on initial CT imaging may be at increased risk of delayed splenic pseudoaneurysm formation.

## Introduction

The spleen is commonly injured in pediatric blunt trauma, with approximately 10% of patients undergoing nonoperative management (NOM) developing delayed splenic pseudoaneurysms (PSAs) [1]. Rupture of these pseudoaneurysms can be life-threatening; thus, careful monitoring and timely intervention using interventional radiology (IVR) are crucial to prevent catastrophic hemorrhage [2,3,4,5].

Despite the clinical significance of delayed PSA formation, standardized follow-up protocols for detecting these lesions have not been established [6,7,8]. Therefore, identifying factors associated with PSA formation is essential to support clinical decision-making. However, these predictive factors remain unclear, leaving clinicians uncertain about whether to proceed with interventional treatment or adopt a watchful waiting approach.

Latent class analysis (LCA) may offer a useful method for identifying sub-phenotypes that predict the development of PSAs following blunt splenic injury [9]. By classifying patients into distinct subgroups based on clinical characteristics, LCA has the potential to provide valuable insights that inform personalized management strategies. Although exploratory in nature, such analyses may lay the groundwork for future studies. Given this background, the objective of our study was to identify sub-phenotypes predictive of delayed PSA formation in pediatric patients with blunt splenic injury using LCA.

## Materials and Methods

### Study Design

This study is a post hoc analysis of a multicenter, retrospective observational study conducted by the Splenic and Hepatic Injury in Pediatric Patients (SHIPPs) study group [1]. The SHIPPs registry compiled pre- and in-hospital data on pediatric patients aged ≤16 years who sustained blunt liver and/or splenic injuries and were admitted to 83 hospitals across Japan between January 2008 and December 2019. Data were entered into a web-based system by medical personnel at each participating institution. Outcome assessors were not blinded to clinical information.

The original SHIPPs study and its subsequent analyses were approved by the Ethics Committee of Jichi Medical University Saitama Medical Center (approval number: S20-112). As this investigation is a secondary analysis of the SHIPPs registry, no additional ethical approval was required. Informed consent was waived, as the study involved no interventions deviating from standard clinical practice; however, an opt-out option was made available on the institution’s website. The data were accessed for research purposes on June 6, 2024. No personally identifiable information was available to the investigators during or after data collection.

This study was conducted in accordance with the STrengthening the Reporting of OBservational Studies in Epidemiology (STROBE) guidelines and the principles outlined in the Declaration of Helsinki and its subsequent amendments (Table S1 in Supplementary Online File 1) [10].

### Participants

The inclusion criteria for the SHIPPs study were as follows: (1) age ≤16 years, (2) transportation by ambulance, (3) diagnosis of splenic and/or liver injury upon hospital arrival, and (4) an Abbreviated Injury Scale (AIS) score ≥1 related to the liver or spleen [1]. The exclusion criteria were: (1) cardiopulmonary arrest on arrival, (2) any injury with an AIS score of 6, (3) duplicate enrollment due to interhospital transfers from both referring and receiving institutions, and (4) refusal of treatment or request for treatment limitation by the parent or guardian due to severe head injury [1].

For the present analysis, additional exclusion criteria were applied: (1) isolated liver injury or combined liver and splenic injuries, (2) cases requiring surgical intervention, (3) pseudoaneurysm formation identified on the day of admission, (4) missing data regarding delayed splenic pseudoaneurysm formation, and (5) transfer to another hospital within 5 days of admission without subsequent follow-up information. In the SHIPPs study, the study cohort was divided into four groups based on hemostatic intervention within 48 hours of admission: NOM (observed), NOM with IVR, operative management (OM), and combined IVR/OM [1]. In contrast, in the current study, we included only cases that fell under NOM (observed), excluding those who underwent surgery or required emergency IVR due to the presence of a splenic pseudoaneurysm at the time of admission.

### Data Collection

The following data were collected: age, sex, weight, past medical history (hematologic disease, neuropsychiatric disorder, chromosomal abnormalities, congenital anomaly, asthma, others), cause of injury (fall, fall down, sport, bicycle, motor vehicle crash, struck by vehicle, abuse, assault, others), vital signs on arrival (heart rate [HR], blood pressure [BP]), presence or absence of shock on arrival, results of blood tests conducted on arrival (hemoglobin [Hb] level, platelet [Plt] count, prothrombin time-international normalized ratio [PT-INR]), initial CT findings (capsular tear of the spleen [none, yes, unknown], presence or absence of contrast extravasation (CE) in the spleen, presence or absence of intra-abdominal bleeding, concomitant injury to other organs), organ injury scaling (OIS) 2018 grade of the spleen on arrival, injury severity score (ISS), blood transfusion, cryoprecipitate transfusion, fibrinogen transfusion, infusion of tranexamic acid, the method of IVR (angiography only, embolization with Gelatin Sponge, embolization with coil, others, unknown), duration of hospitalization, 30-day mortality, hospital mortality, and formation of delayed splenic pseudoaneurysm [11,12].

Shock was defined according to the Pediatric Advanced Life Support (PALS) guidelines of the American Heart Association [13]. For transfusion, distinctions were not made between the types of red blood cells (RBCs), fresh frozen plasma (FFP), or Plt.

### Outcome Measure

The outcome measure was delayed splenic pseudoaneurysm formation. This outcome measure was defined as the formation of a splenic pseudoaneurysm, which was detected on or after the second day after injury (but undetectable on CT scan on admission). The determination of whether delayed splenic pseudoaneurysm had formed was left to the discretion of the physicians at each facility.

### Statistical Analyses

We selected the following clinically relevant variables for latent class analysis (LCA): age, sex, weight, past medical history, mechanism of injury, presence or absence of shock on arrival, blood test results at admission, initial CT findings, splenic injury grade based on the 2018 Organ Injury Scale (OIS), Injury Severity Score (ISS), blood transfusion during hospitalization, administration of cryoprecipitate, fibrinogen transfusion, tranexamic acid infusion, and the type of IVR procedure performed. Multiple imputation of explanatory variables was conducted using the Markov chain Monte Carlo method. Given that the maximum proportion of missing data was 24%, a total of 24 imputed datasets were generated [14]. Although the exact timing of transfusions was not recorded, we assumed they were administered during the acute phase immediately after injury—prior to the potential formation of delayed splenic pseudoaneurysms.

We initially explored models with 2 to 5 latent classes and determined the optimal number of clinically meaningful sub-phenotypes based on both the Bayesian Information Criterion (BIC) and clinical interpretability. After identifying the optimal class structure, the discriminative capacity of each variable was evaluated using the maximum integrated complete-data likelihood criterion. Higher variable indices indicated stronger associations between the variables and class membership.

After sub-phenotypes were established, continuous variables were summarized as medians and interquartile ranges (IQRs), while categorical variables were reported as absolute numbers and percentages. Differences in variables among the latent classes were assessed using Pearson’s chi-squared test or the Kruskal–Wallis rank sum test, as appropriate. Logistic regression analysis was subsequently performed to examine the association between each sub-phenotype and the occurrence of delayed splenic pseudoaneurysms, expressed as odds ratios (ORs) with corresponding 95% confidence intervals (CIs). No covariate adjustment was performed, as the latent classes were conceptualized as unobserved homogeneous subgroups based on patient characteristics, and thus covariates were not considered to confound the relationship between latent classes and outcomes [15].

All analyses were performed using the R package *VarSelLCM* and R statistical software (version 4.1.3; The R Project for Statistical Computing, Vienna, Austria). A two-sided p-value of <0.05 was considered statistically significant.

## Results

### Patient Enrollment

**Fig. 1.**
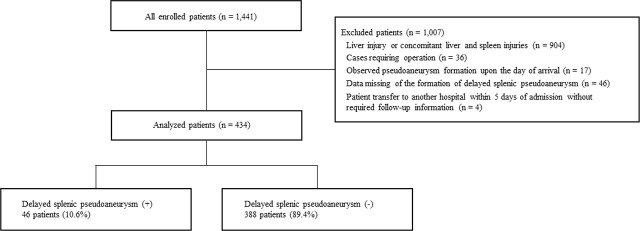
Flowchart depicting the screening and enrollment process in this study

Of the 1,441 patients included in the registry of pediatric hepatic and splenic injuries, we analyzed the data of 434 patients who met the inclusion criteria for the current study (Figure 1).

### LCA

Three Sub-phenotypes were identified after LCA (Table S2 in Supplementary Online File 2). Patient characteristics and pre- and post-hospital information for each latent class are listed in Table 1, and missing values are shown in Table S3 in Supplementary Online file 2.

**Fig. 2.**
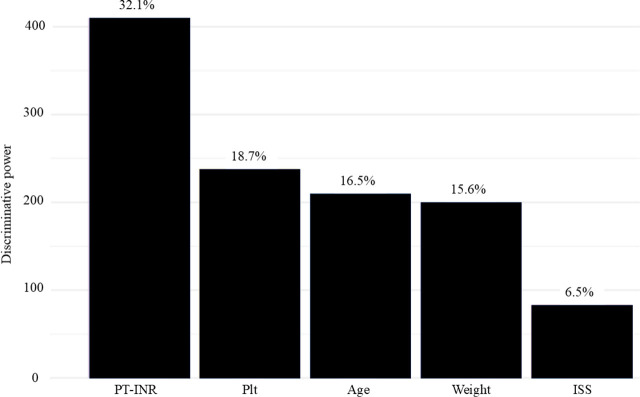
**The five factors showing high discriminative power in LCA.** Abbreviations: ISS, Injury Severity Scale score; LCA, latent class analysis; Plt, platelet count; PT-INR, prothrombin time-international normalized ratio.

The factor with the highest discriminative power was PT-INR (Figure 2). The following values are all listed in the order of Sub-phenotype 1, Sub-phenotype 2, and Sub-phenotype 3. The median (IQR) values of PT-INR were as follows: 1.27 (1.18–1.56), 1.15 (1.08–1.24), and 1.08 (1.02–1.14); and plt on arrival were as follows: 22.8 (15.7–28.1) ×10^3^/μL, 23.9 (20.8–28.7) ×10^3^/μL, and 28.9 (23.1–33.3) ×10^3^/μL (Table 1, and Figure S1-a and S1-b in Supplementary Online File 2). Among the other variables, shock was observed in six patients (18.8%), eight patients (4.2%), and five patients (2.4%); CE in the spleen of initial CT was observed in 22 patients (68.8%), 49 patients (25.7%), and 22 patients (10.4%); OIS grade 4 or 5 for the spleen was observed in 19 patients (59.4%), 44 patients (23.0%), and 31 patients (14.7%); the median (IQR) values of ISS were as follows: 31.5 (21.5–42.3), 9 (5.5–17), and 9 (5–14); and blood transfusion was performed in 25 patients (78.1%), 25 patients (13.1%), and 19 patients (9.0%) (Table 1, and Figure S1-c–S1-e in Supplementary Online File 2). And the variables discussed above, although not ranked among those with the highest discriminative power, are presented in Table S4 in Supplementary Online File 2. Furthermore, the variables with statistically significant differences among the LCA-derived sub-phenotypes are shown in Table S5 in Supplementary Online File 2.

**Table 1.** Demographics and characteristics of analyzed patients with stratified by sub-phenotypes

**Variables**	**Overall (n=434) Formation of delayed splenic pseudoaneurysm: n=46 (10.6%)**	**Sub-phenotypes**			**p value**

		**Sub-phenotype 1 (n=32) Formation of delayed splenic pseudoaneurysm: n=7 (21.9%)**	**Sub-phenotype 2 (n=191) Formation of delayed splenic pseudoaneurysm: n=25 (13.1%)**	**Sub-phenotype 3 (n=211) Formation of delayed splenic pseudoaneurysm: n=14 (6.6%)**	
Age, years, median (IQR)	10 (7–13)	9 (7.8–12)	13 (12–15)	8 (5.5–9)	< .001
Male, n (%)	303 (69.8)	19 (59.4)	145 (75.9)	139 (65.9)	0.03
Weight, kg, median (IQR)	33 (24.1–47)	29.5 (25–39.8)	48 (41–55)	25 (19.4–29.5)	< .001

Past medical history, n (%)					
None	366 (84.3)	30 (93.8)	153 (80.1)	183 (86.7)	0.65
Hematologic disease	2 (0.5)	0 0)	1 (0.5)	1 (0.5)	
Neuropsychiatric disorder	23 (5.3)	0 (0)	13 (6.8)	10 (4.7)	
Chromosomal abnormalities	0 (0)	0 (0)	0 (0)	0 (0)	
Congenital anomaly	5 (1.2)	0 (0)	2 (1.0)	3 (1.4)	
Asthma	22 (5.1)	1 (3.1)	14 (7.3)	7 (3.3)	
Others	16 (3.7)	1 (3.1)	8 (4.2)	7 (3.3)	

Situation of injury, n (%)					
Fall	109 (25.1)	6 (18.8)	29 (15.2)	74 (35.1)	0.009
Fall down	41 (9.5)	2 (6.2)	10 (5.2)	29 (13.7)	
Sport	66 (15.2)	2 (6.2)	50 (26.2)	14 (6.6)	
Bicycle	82 (18.9)	5 (15.6)	53 (27.7)	24 (11.4)	
Motor vehicle crash	49 (11.3)	7 (21.9)	21 (11.0)	21 (10.0)	
Struck by vehicle	59 (13.6)	9 (28.1)	11 (5.8)	39 (18.5)	
Abuse	1 (0.2)	0 (0)	0 (0)	1 (0.5)	
Assault	12 (2.8)	1 (3.1)	11 (5.8)	0 (0)	
Others	15 (3.5)	0 (0)	6 (3.1)	9 (4.3)	
Shock on arrival, n (%)	19 (4.4)	6 (18.8)	8 (4.2)	5 (2.4)	0.002
Hb on arrival, mg/dl, median (IQR)	12.3 (11.2–13.3)	11.1 (8.8–11.9)	13.1 (11.7–13.9)	12.0 (11.2–12.8)	< .001
Plt on arrival, ×103/μL, median (IQR)	25.7 (21.6–31.3)	22.8 (15.7–28.1)	23.9 (20.8–28.7)	28.9 (23.1–33.3)	< .001
PT-INR on arrival, median (IQR)	1.12 (1.05–1.21)	1.27 (1.18–1.56)	1.15 (1.08–1.24)	1.08 (1.02–1.14)	< .001

Capsular tear of spleen, n (%)					
None	118 (27.2)	2 (6.2)	53 (27.7)	63 (29.9)	0.03
Yes	298 (68.7)	30 (93.8)	131 (68.6)	137 (64.9)	
Unknown	18 (4.2)	0 (0)	7 (3.7)	11 (5.2)	
Contrast extravasation in spleen, n (%)	93 (21.4)	22 (68.8)	49 (25.7)	22 (10.4)	< .001
Intra-abdominal bleeding, n (%)	355 (81.8)	28 (87.5)	158 (82.7)	169 (80.1)	0.60
Concomitant injury to other organs, n (%)	136 (31.3)	20 (62.5)	65 (34.0)	51 (24.2)	< .001

OIS 2018 of spleen, n (%)					
1	34 (7.8)	1 (3.1)	12 (6.3)	21 (10.0)	< .001
2	158 (36.4)	4 (12.5)	71 (37.2)	83 (39.3)	
3	148 (34.1)	8 (25.0)	64 (33.5)	76 (36.0)	
4	73 (16.8)	15 (46.9)	34 (17.8)	24 (11.4)	
5	21 (4.8)	4 (12.5)	10 (5.2)	7 (3.3)	
ISS, median (IQR)	9 (6–17)	31.5 (21.5–42.3)	9 (5.5–17)	9 (5–14)	
Blood transfusion, n (%)	69 (15.9)	25 (78.1)	25 (13.1)	19 (9.0)	
Cryoprecipitate transfusion, n (%)	4 (0.9)	3 (9.4)	0 (0)	1 (0.5)	0.001
Fibrinogen transfusion, n (%)	2 (0.5)	0 (0)	1 (0.5)	1 (0.5)	1.0
Infusion of tranexamic acid, n (%)	95 (21.9)	8 (25.0)	50 (26.2)	37 (17.5)	0.09

Method of IVR					
None or angiography only	425 (97.9)	31 (96.9)	185 (96.9)	209 (99.1)	0.28
Embolization with Gelatin Sponge	5 (1.2)	0 (0)	4 (2.1)	1 (0.5)	
Embolization with coil	4 (0.9)	1 (3.1)	2 (1.0)	1 (0.5)	
Others	0 (0)	0 (0)	0 (0)	0 (0)	
Unknown	0 (0)	0 (0)	0 (0)	0 (0)	

Abbreviations: Hb, hemoglobin; ISS, injury severity score; IQR, interquartile range; IVR, interventional radiology; OIS, Organ Injury Scale; Plt, platelet; PT-INR, prothrombin time-international normalized ratio.

### Sub-phenotypes and Delayed Splenic Pseudoaneurysm Formation

**Fig. 3.**
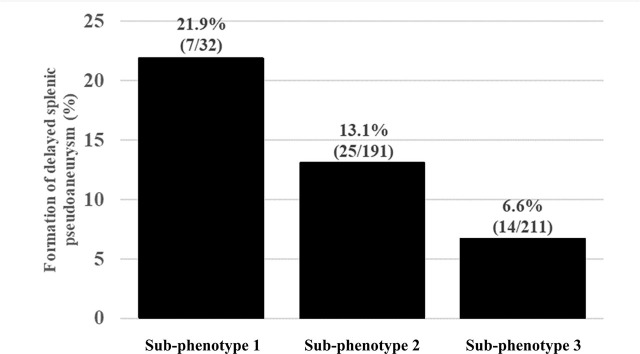
Formation of delayed splenic pseudoaneurysm in each Sub-phenotype

In total, delayed splenic pseudoaneurysm formation was observed in 46 patients (10.6%), including seven patients (21.9%) in Sub-phenotype 1, 25 patients (13.1%) in Sub-phenotype 2, and 14 patients (6.6%) in Sub-phenotype 3 (p = 0.01; Table 2 and Figure 3).

**Table 2.** Sub-phenotypes and delayed splenic pseudoaneurysm formation

	**Overall (n=434)**	**Sub-phenotypes**			**p value**

		**Sub-phenotype 1 (n=32)**	**Sub-phenotype 2 (n=191)**	**Sub-phenotype 3 (n=211)**	
Formation of delayed splenic pseudoaneurysm, n (%)	46 (10.6)	7 (21.9)	25 (13.1)	14 (6.6)	0.01

### Logistic Regression Analysis

Univariate logistic regression analysis for delayed splenic pseudoaneurysm formation using Sub-phenotype 3 as the reference revealed an OR (95% CI) of 3.94 (1.45–10.7) in Sub-phenotype 1 and 2.12 (1.07–4.21) in Sub-phenotype 2 (Table 3).

**Table 3.** Logistic regression analysis for the correlation between sub-phenotypes and delayed splenic pseudoaneurysm formation

	**Crude OR**	**95% CI (lower)**	**95% CI (upper)**	**p value**
Formation of delayed splenic pseudoaneurysm				
Sub-phenotypes				
Sub-phenotype 1	3.94	1.45	10.7	0.007
Sub-phenotype 2	2.12	1.07	4.21	0.03
Sub-phenotype 3	ref	ref	ref	-

Abbreviations: CI, confidence interval; OR, odds ratio.

## Discussion

The current LCA revealed three Sub-phenotypes. Statistically significant differences were observed in the distribution of shock, CE in the spleen, OIS 2018 grade 4 or 5 for the spleen, blood transfusion, cryoprecipitate transfusion, and delayed splenic pseudoaneurysm formation among these Sub-phenotypes. Clinically, patients with severe splenic injury—such as OIS 2018 grade 4 or 5—or those with CE in the spleen and a high ISS often present with shock due to massive hemorrhage resulting from complete vascular disruption. These cases are frequently associated with coagulopathy, including prolonged PT-INR and thrombocytopenia, and typically require blood transfusion as well as cryoprecipitate administration. In such patients, particular attention should be paid to the potential development of delayed splenic pseudoaneurysm formation.

In the current LCA, shock, CE in the spleen, OIS 2018 grade 4 or 5 for the spleen, ISS, blood transfusion, and cryoprecipitate transfusion were significantly higher in Sub-phenotype 1. Additionally, Sub-phenotype 1 was characterized by a significantly prolonged PT-INR and a decreased platelet count. Moreover, a spleen OIS 2018 grade of 4 or 5 is associated with a higher AIS score for the spleen, which consequently tends to lead to an increased ISS. The CE and OIS 2018 grade 4 or 5 of the spleen suggested complete disruption of the arterial wall. And pseudoaneurysms result from complete disruption of the arterial wall, such as trauma [16]. Therefore, in the current LCA, complete disruption of the splenic arterial wall due to trauma may result in delayed splenic pseudoaneurysm formation. And blood leakage caused by complete disruption of the splenic arterial wall leads to a reduction in circulating blood volume and subsequent shock. Bleeding consumes platelets and coagulation factors, resulting in prolonged PT-INR. However, with respect to age and body weight, we were unable to identify a consistent pattern between their distribution across the three sub-phenotypes and the frequency of delayed splenic pseudoaneurysm formation. Therefore, it was challenging to provide a logical interpretation of our LCA-derived sub-phenotypes based on these factors.

To the best of our knowledge, no previous studies have proposed predictive models for the formation of delayed splenic pseudoaneurysms following NOM of blunt splenic trauma in pediatric patients. Although predictive modeling has not been extensively explored, several studies have reported risk factors associated with delayed PSA formation, recognizing that rupture of such lesions can be life-threatening [3,17]. In particular, these studies have identified a correlation between CE on initial CT and subsequent formation or rupture of delayed splenic pseudoaneurysms [3,17]. While these prior investigations were observational in nature and warrant cautious interpretation, their findings are consistent with those of the present LCA, suggesting the robustness of our results [3,17].

The novelty of the current study lies in its application of an LCA-based approach to identify sub-phenotypes predictive of delayed PSA formation in pediatric patients undergoing NOM for blunt splenic injury—an area that remains underexplored. Our findings suggest that cases demonstrating CE on initial CT imaging may warrant close inpatient observation and follow-up imaging due to the potential risk of delayed pseudoaneurysm formation. Conversely, in the absence of CE, unnecessary hospitalization and follow-up imaging may be avoidable. Nonetheless, due to the low incidence of delayed pseudoaneurysm formation, the clinical applicability of the current LCA findings should be interpreted with caution. However, as noted in the SHIPPs study, which served as the foundation for the current study, we believe that our findings offer several important clinical implications regarding splenic pseudoaneurysm formation following blunt splenic injury in pediatric patients. First, given the current paucity of research in this area, particularly in the pediatric population, our data of the current study may serve as a valuable basis for future investigations. Second, in countries such as Japan, where standardized guidelines have yet to be established, our findings may provide an opportunity to reevaluate existing clinical practices and potentially inform future decision-making regarding the management of pediatric blunt splenic injuries.

This study has several limitations. First, the findings may lack external validity. Given the relatively small sample size and the absence of standardized criteria for sample size determination in LCA, the development of a validation cohort and corresponding LCA was not feasible. Furthermore, the low incidence of delayed pseudoaneurysm formation precluded the use of resampling techniques such as bootstrap validation, limiting the generalizability of our results to broader populations. Second, the clinical utility of the three sub-phenotypes identified in this study may be constrained by the rarity of the outcome. Previous research has suggested that a higher overall event rate is associated with improved class separation in LCA, which may not have been fully achievable in our dataset [18]. In the current study, three sub-phenotypes showed statistically significant differences in the incidence rates of delayed splenic pseudoaneurysms. However, with the highest incidence rate being 21.9% in Sub-phenotype 1, this percentage is too low to be clinically useful for predicting the occurrence of delayed splenic pseudoaneurysms. Therefore, although the results of the current study are statistically significant, their clinical utility might be limited. Finally, since we excluded cases of combined blunt liver and spleen injuries, it is possible that we did not target a high-risk patient group for the formation of delayed splenic pseudoaneurysms. Previous studies have reported that combined blunt liver and spleen injuries are more severe and exhibit a higher incidence of CE compared to isolated spleen injuries [19]. Therefore, the current study might have excluded high-risk patients for delayed splenic pseudoaneurysms formation, potentially leading to inaccurate analysis results.

## Conclusions

We performed LCA for the formation of delayed splenic pseudoaneurysms in pediatric patients with blunt splenic trauma and underwent NOM. We classified the patients into three sub-phenotypes showing statistically significant differences related to the formation of delayed splenic pseudoaneurysms. Our findings suggest that cases with CE on initial CT imaging may be at increased risk of delayed splenic pseudoaneurysm formation. However, owing to the low incidence rates of delayed splenic pseudoaneurysm formation, the clinical use of these three sub-phenotypes may be limited.

## References

[1] Katsura M, Kondo Y, Yasuda H, Fukuma S, Matsushima K, Shiraishi A (2023). Therapeutic strategies for pseudoaneurysm following blunt liver and spleen injuries: A multicenter cohort study in the pediatric population. J Trauma Acute Care Surg..

[2] Safavi A, Beaudry P, Jamieson D, Murphy JJ (2011). Traumatic pseudoaneurysms of the liver and spleen in children: is routine screening warranted. J Pediatr Surg..

[3] Katsura M, Fukuma S, Kuriyama A, Takada T, Ueda Y, Asano S (2020). Association between contrast extravasation on computed tomography scans and pseudoaneurysm formation in pediatric blunt splenic and hepatic injury: A multi-institutional observational study. J Pediatr Surg..

[4] Gates RL, Price M, Cameron DB, Somme S, Ricca R, Oyetunji TA (2019). Non-operative management of solid organ injuries in children: An American Pediatric Surgical Association Outcomes and Evidence Based Practice Committee systematic review. J Pediatr Surg..

[5] Swendiman RA, Goldshore MA, Fenton SJ, Nance ML (2020). Defining the role of angioembolization in pediatric isolated blunt solid organ injury. J Pediatr Surg..

[6] van der Vlies CH, Saltzherr TP, Wilde JCH, Delden OM, de Haan RJ, Goslings JC (2010). The failure rate of nonoperative management in children with splenic or liver injury with contrast blush on computed tomography: a systematic review. J Pediatr Surg.

[7] Ingram M-CE, Siddharthan RV, Morris AD, Hill SJ, Travers CD, McKracken CE (2016). Hepatic and splenic blush on computed tomography in children following blunt abdominal trauma: is intervention necessary?. J Trauma Acute Care Surg.

[8] Bansal S, Karrer FM, Hansen K, Partrick DA (2015). Contrast blush in pediatric blunt splenic trauma does not warrant the routine use of angiography and embolization. Am J Surg.

[9] Lanza ST, Rhoades BL (2013). Latent class analysis: an alternative perspective on subgroup analysis in prevention and treatment. Prev Sci..

[10] von Elm E, Altman DG, Egger M, Pocock SJ, Gøtzsche PC, Vandenbroucke JP (2014). The Strengthening the Reporting of Observational Studies in Epidemiology (STROBE) Statement: guidelines for reporting observational studies. Int J Surg..

[11] Kozar RA, Crandall M, Shanmuganathan K, Zarzaur BL, Coburn M, Cribari C (2018). Organ injury scaling 2018 update: Spleen, liver, and kidney. J Trauma Acute Care Surg..

[12] Baker SP, O’Neill B, Haddon W, Long WB (1974). The injury severity score: a method for describing patients with multiple injuries and evaluating emergency care. J Trauma..

[13] Kleinman ME, Chameides L, Schexnayder SM, Samson RA, Hazinski MF, Atkins DL (2010). Pediatric advanced life support: 2010 American Heart Association Guidelines for Cardiopulmonary Resuscitation and Emergency Cardiovascular Care. Pediatrics..

[14] Hayati Rezvan P, Lee KJ, Simpson JA (2015). The rise of multiple imputation: a review of the reporting and implementation of the method in medical research. BMC Med Res Methodol..

[15] Okada Y, Komukai S, Kitamura T, Kiguchi T, Irisawa T, Yamada T (2022). Clinical Phenotyping of Out-of-Hospital Cardiac Arrest Patients With Shockable Rhythm- Machine Learning-Based Unsupervised Cluster Analysis. Circ J..

[16] Sousa J, Costa D, Mansilha A (2019). Visceral artery aneurysms: review on indications and current treatment strategies. Int Angiol..

[17] Skattum J, Gaarder C, Naess PA (2014). Splenic artery embolisation in children and adolescents--an 8 year experience. Injury.

[18] Gudicha DW, Schmittmann VD, Vermunt JK (2017). Statistical power of likelihood ratio and Wald tests in latent class models with covariates. Behav Res Methods..

[19] Fodor M, Primavesi F, Morell-Hofert D, Kranebitter V, Palaver A, Braunwarth E (2019). Non-operative management of blunt hepatic and splenic injury: a time-trend and outcome analysis over a period of 17 years. World J Emerg Surg..

